# Supplementary figures and images for: SPARK regulates AGC kinases central to the Toxoplasma gondii asexual cycle
Source: eLife. 2024 Aug 13;13:RP93877. doi: 10.7554/eLife.93877 (PMC11321763; doi:10.7554/eLife.93877)

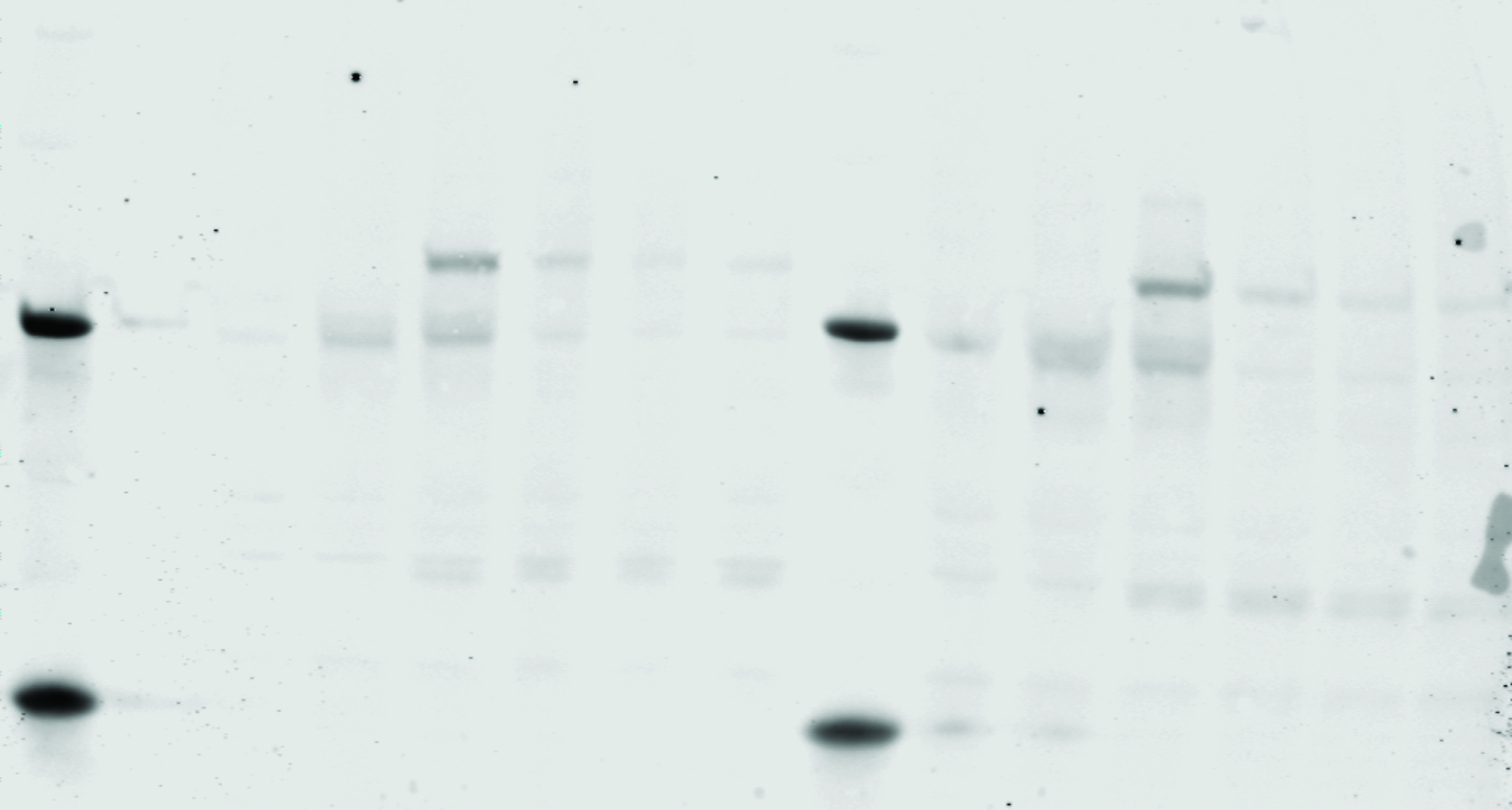

Supplement: Figure 2—source data 1. — V5, LICOR. [file elife-93877-fig2-data1.zip › Figure 2ΓÇösource data 1.tif]

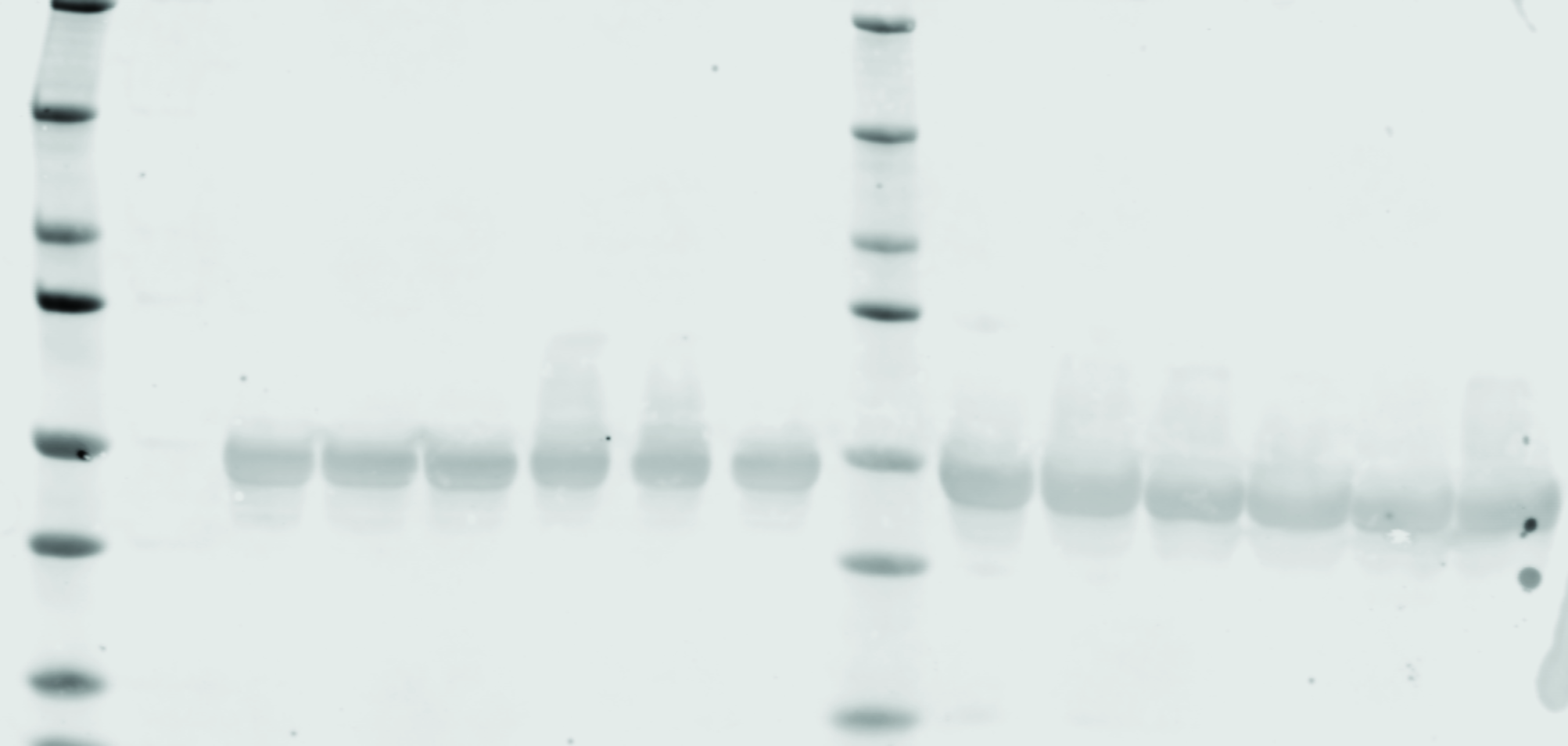

Supplement: Figure 2—source data 2. — TUB1, LICOR. [file elife-93877-fig2-data2.zip › Figure 2ΓÇösource data 2.tif]

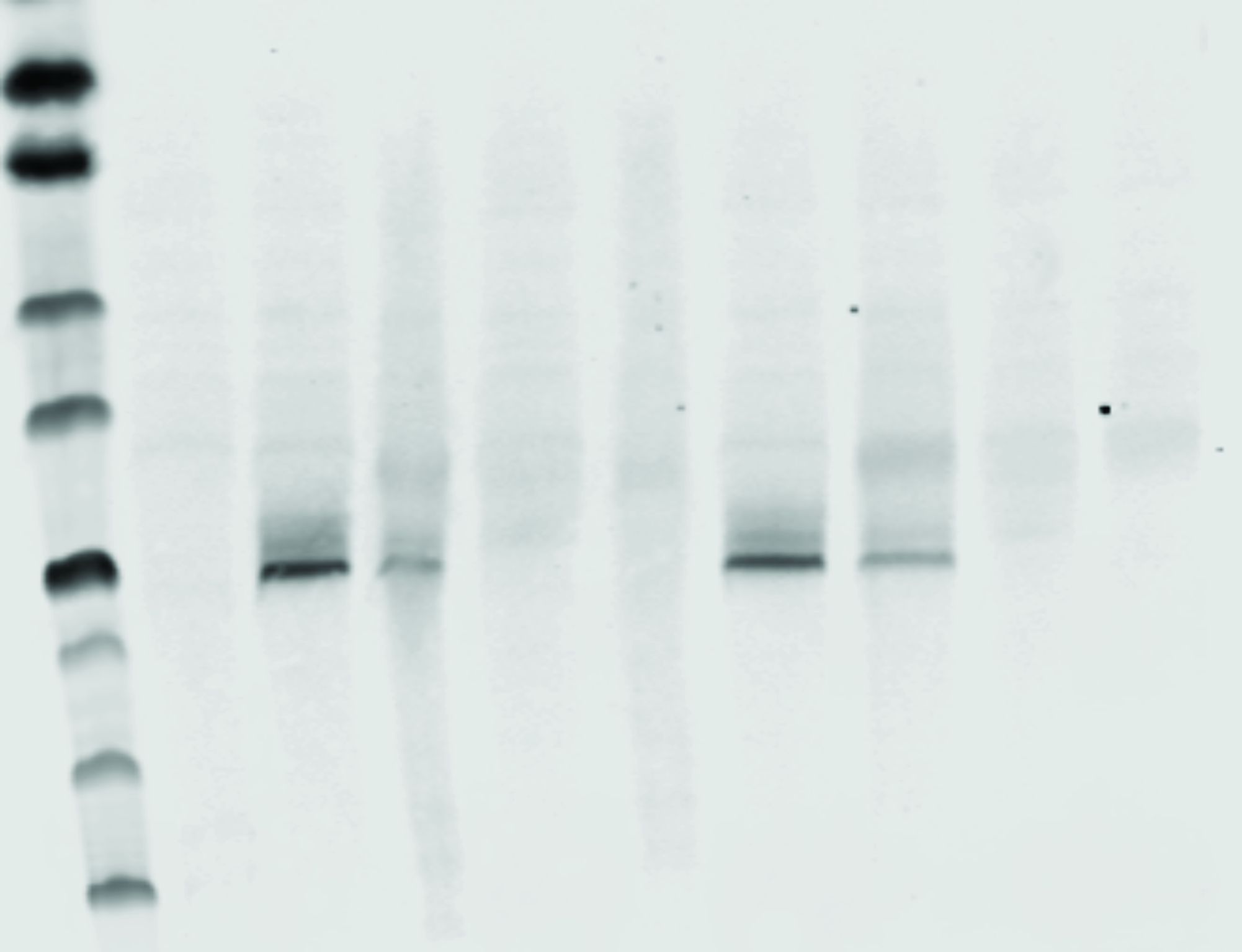

Supplement: Figure 2—source data 3. — V5, LICOR. [file elife-93877-fig2-data3.zip › Figure 2ΓÇösource data 3.tif]

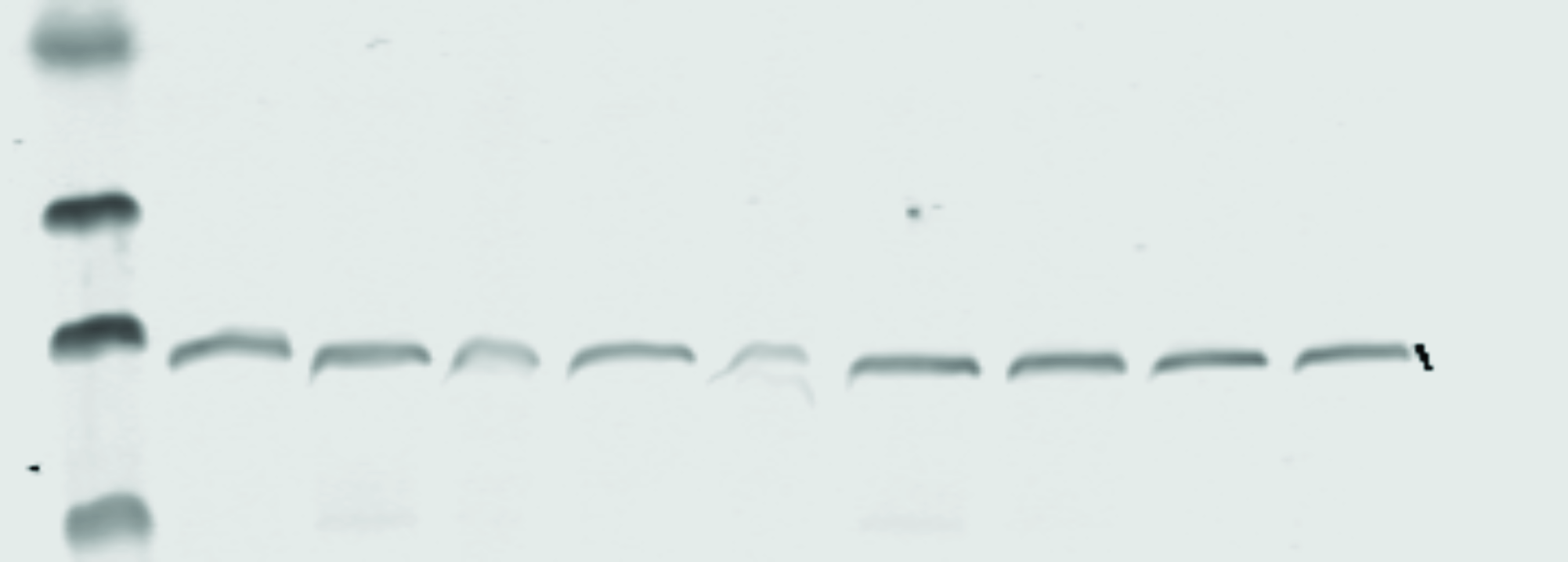

Supplement: Figure 2—source data 4. — CDPK1, LICOR. [file elife-93877-fig2-data4.zip › Figure 2ΓÇösource data 4.tif]

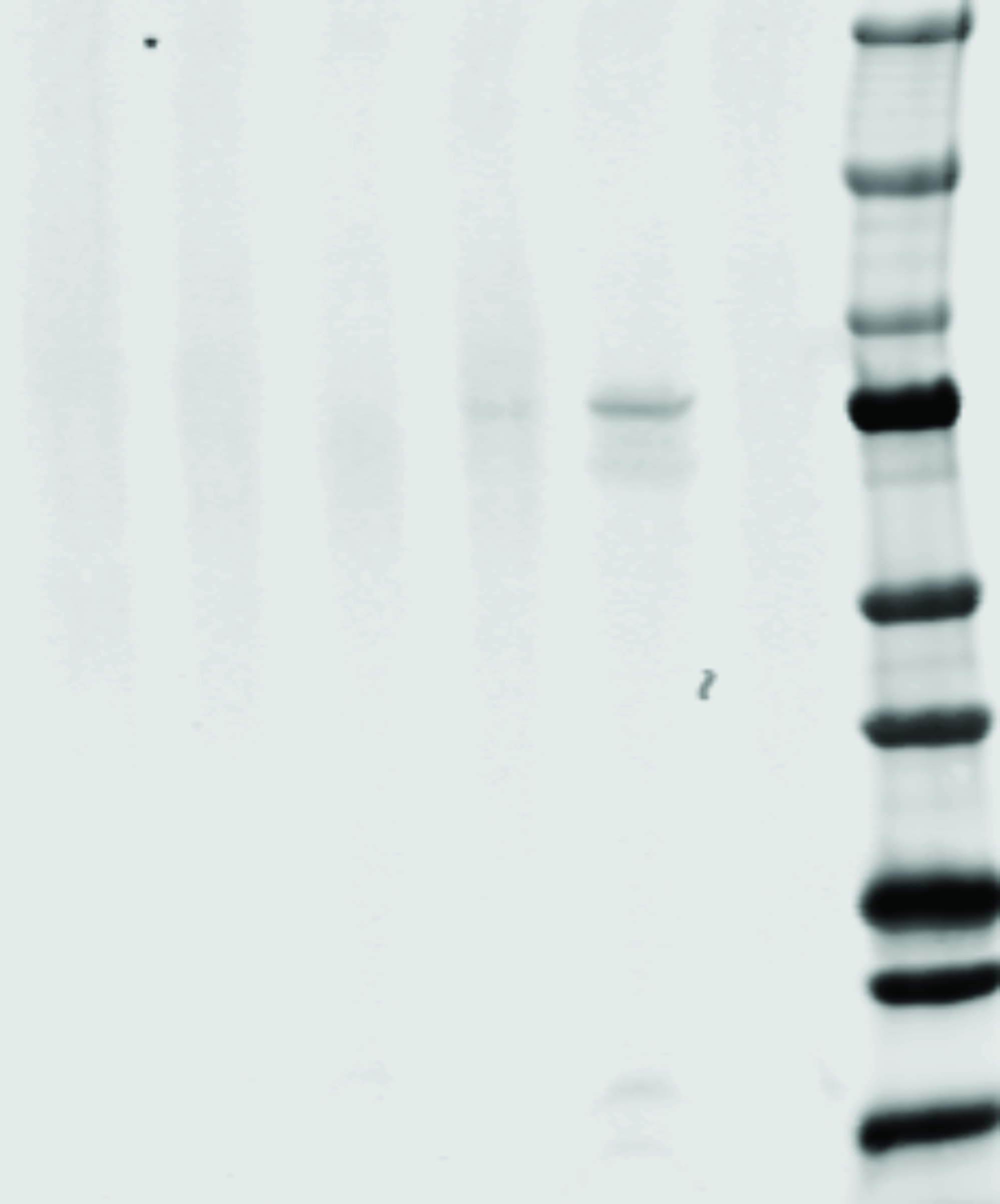

Supplement: Figure 2—figure supplement 1—source data 1. — V5, LICOR. [file elife-93877-fig2-figsupp1-data1.zip › Figure 2ΓÇöfigure supplement 1 source data 1.tif]

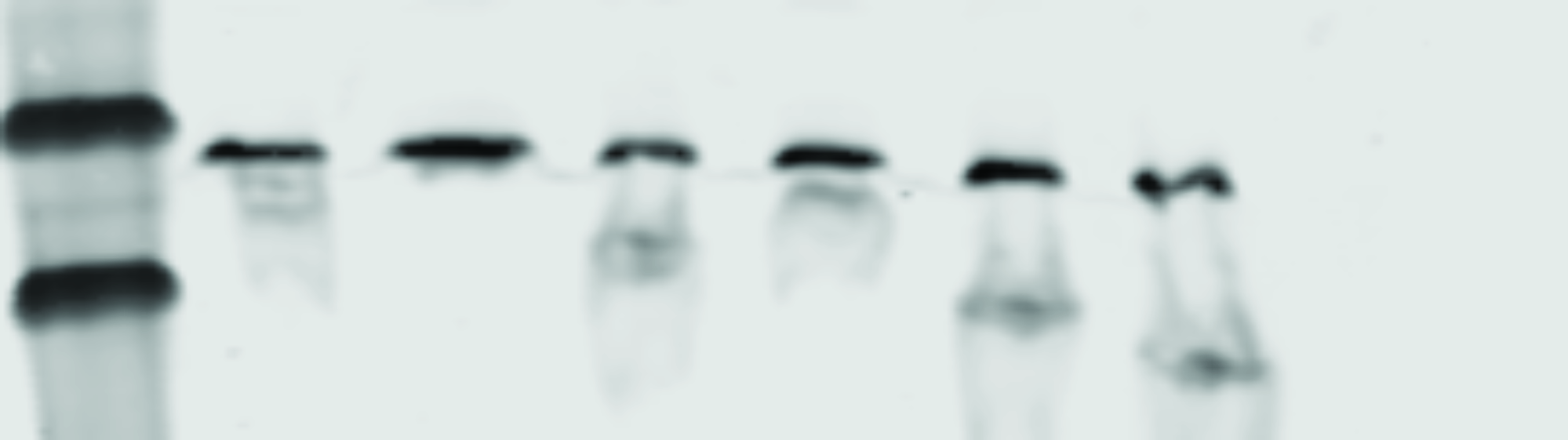

Supplement: Figure 2—figure supplement 1—source data 2. — ALD1, LICOR. [file elife-93877-fig2-figsupp1-data2.zip › Figure 2ΓÇöfigure supplement 1 source data 2.tif]

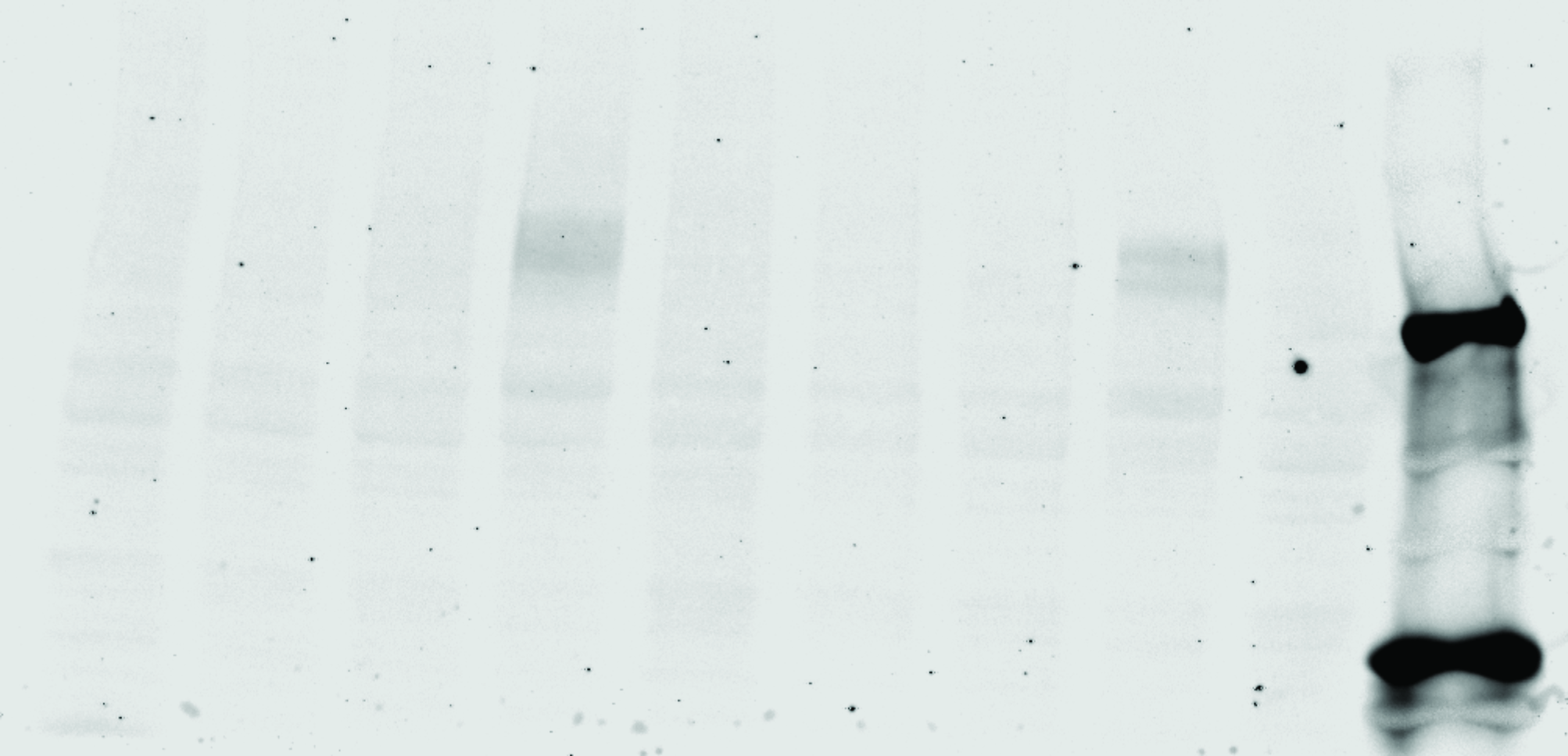

Supplement: Figure 6—source data 1. — V5, LICOR. [file elife-93877-fig6-data1.zip › Figure 6ΓÇösource data 1.tif]

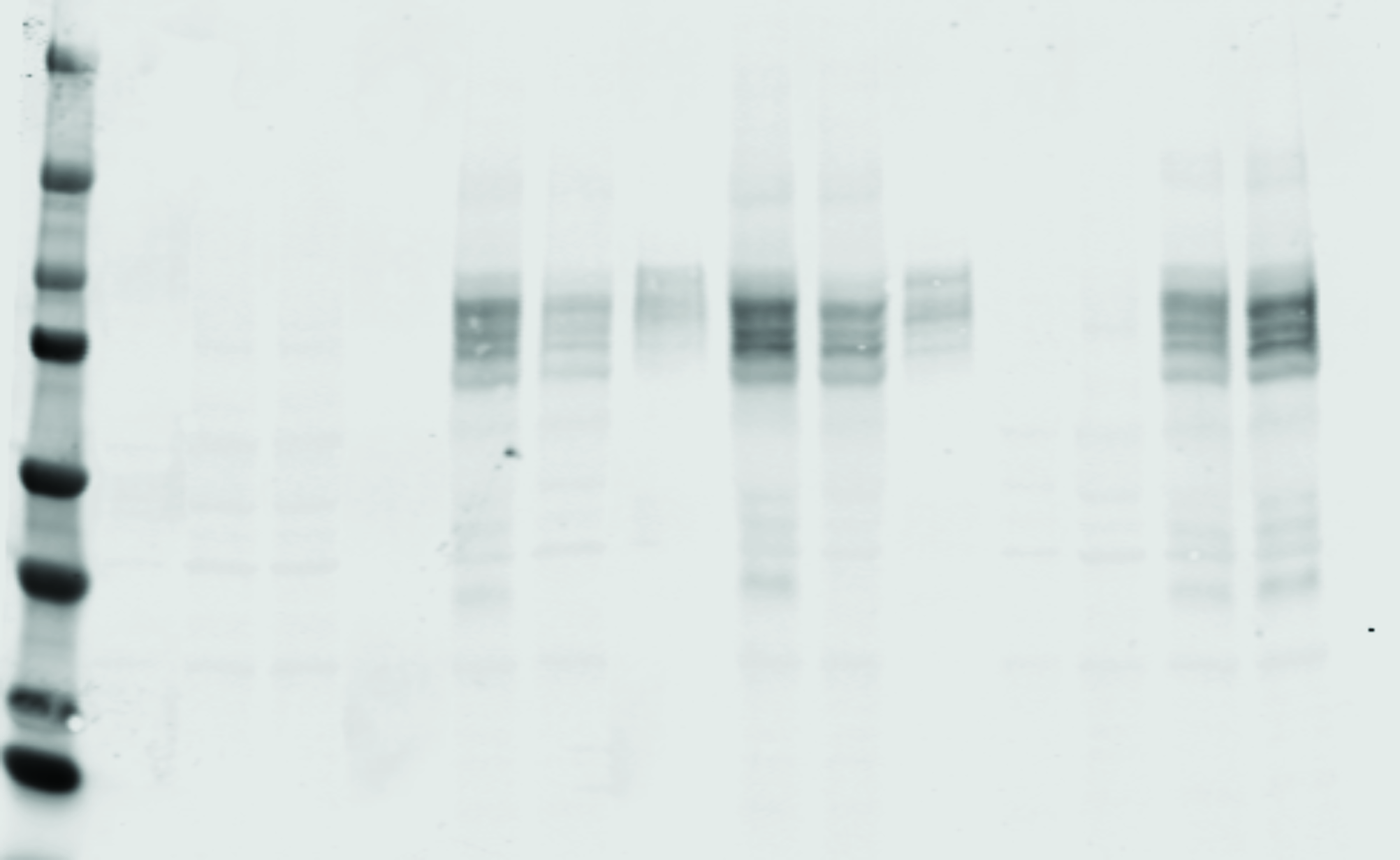

Supplement: Figure 7—source data 1. — mNG, LICOR. [file elife-93877-fig7-data1.zip › Figure 7ΓÇösource data 1.tif]

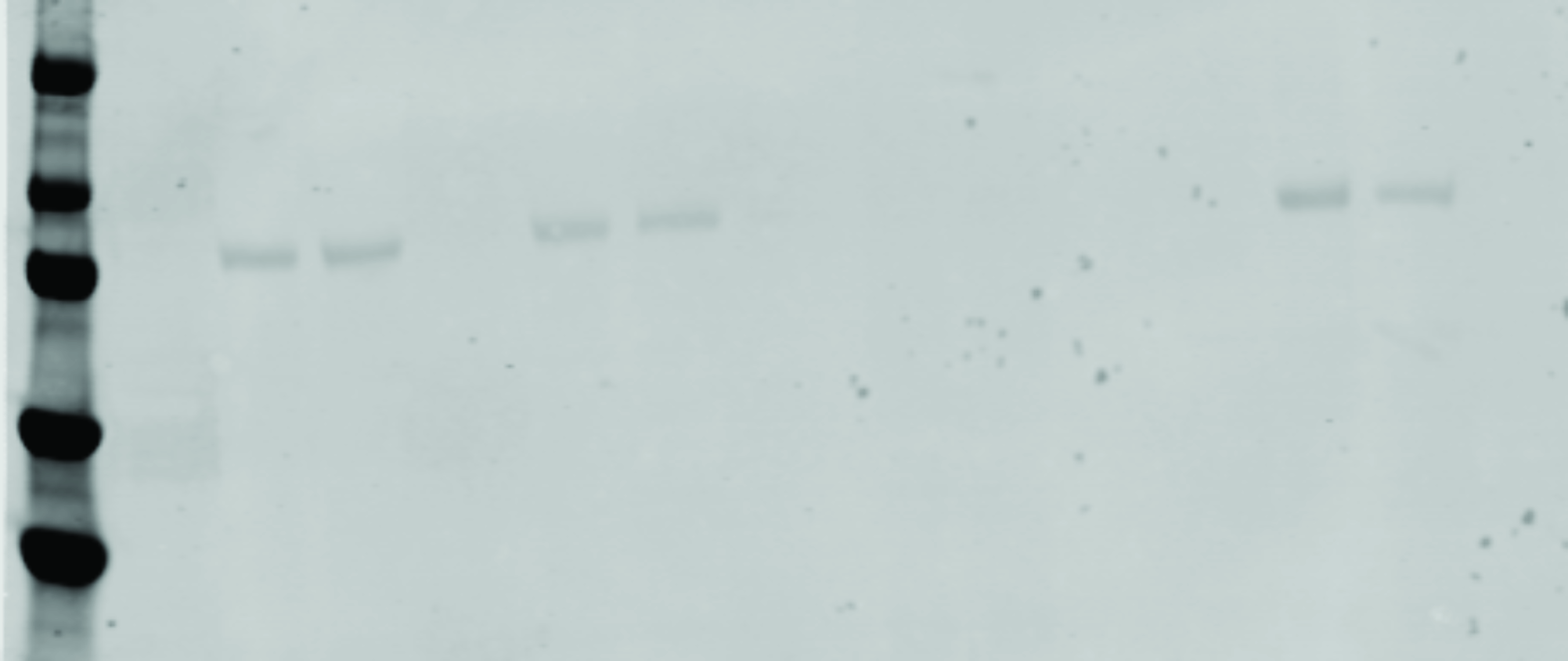

Supplement: Figure 7—source data 2. — V5, LICOR. [file elife-93877-fig7-data2.zip › Figure 7ΓÇösource data 2.tif]

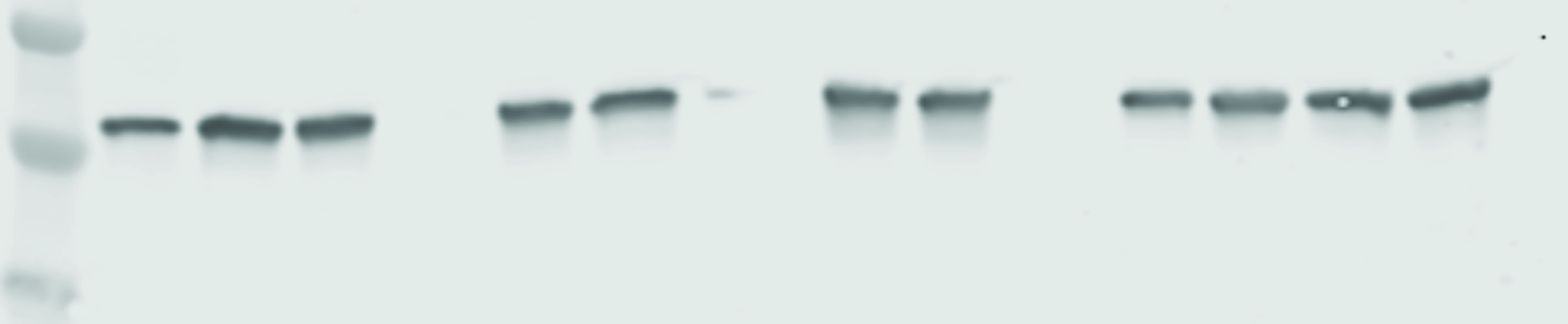

Supplement: Figure 7—source data 3. — ALD1, LICOR. [file elife-93877-fig7-data3.zip › Figure 7ΓÇösource data 3.tif]
